# Joint Recommendations From The Latin American Association of Cardiac and Endovascular Surgery (LACES) and The Cardiovascular Anesthesia Committee of The Latin American Confederation of Anesthesia Societies (CLASA) on the Timing for Cardiac Surgery After COVID-19 Infection

**DOI:** 10.21470/1678-9741-2022-0198

**Published:** 2022

**Authors:** Victor Dayan, Pablo Straneo, Mario Jose Arguello, Mayra Vaca, Luis Eduardo Enriquez, Gunther Krogh, Carlos Alberto Carcausto Humani, Milton Patricio Chango Iza, Ezequiel Leonel Fernandez, Rosina Ruiz Roque, Xavier Mantilla Pinto, Rosemberg Albores Figueroa, Oscar Felipe Heilbron, Marcos Schioppi, Bruno Bismark Camacho Alvarez, Mateo Marin-Cuartas, Gomes Walter J., Juan Riva

**Affiliations:** 1 Department of Cardiac Surgery, Centro Cardiovascular Universitario, Universidad de la República del Uruguay, Montevideo, Uruguay; 2 Department of Cardiac Surgery, Sanatorio Británico, Rosario, Argentina; 3 Department of Anesthesia, Hospital Calderón Guardia, San José, Costa Rica; 4 Department of Anesthesia, Clinica Imbanaco Quirónsalud, Cali, Colombia; 5 Department of Cardiac Surgery, Pontificia Universidad Católica de Chile, Santiago de Chile, Chile; 6 Department of Cardiac Surgery, Clínica San Felipe, Lima, Peru; 7 Department of Anesthesia, Hospital Vozandes, Quito, Ecuador; 8 Department of Cardiac Surgery, Instituto Cardiovascular de Buenos Aires, Buenos Aires, Argentina; 9 Department of Anesthesia, Instituto Nacional Cardiovascular (INCOR-EsSalud), Lima, Peru; 10 Department of Anesthesia, Hospital Metropolitano, Pontificia Universidad Católica del Ecuador, Quito, Ecuador; 11 Department of Anesthesia, Hospital de Cardiología, Monterrey, Mexico; 12 Department of Cardiac Surgery, Clinica PortoAzul, Barranquilla, Colombia; 13 Department of Cardiac Surgery, Instituto Cardiológico Infantil, Montevideo, Uruguay; 14 Department of Cardiac Surgery, Centro Médico Nacional de Occidente, Instituto Mexicano del Seguro Social, Guadalajara, Mexico; 15 Department of Cardiac Surgery, Leipzig Heart Center, Leipzig, Germany; 16 Cardiovascular Surgery Discipline and Hospital São Paulo, Escola Paulista de Medicina, Universidade Federal de São Paulo, São Paulo, São Paulo, Brazil; 17 Department of Anesthesia, Facultad de Medicina, Universidad de la República, Montevideo, Uruguay

**Keywords:** Health Services Needs and Demand, COVID-19, Data Collection, Thoracic Surgery, Severe Acute Respiratory Syndrome Coronavirus 2, Latin America, Vaccination, Time, Waiting, List

## Abstract

**Introduction:**

Since the coronavirus disease 2019 (COVID-19) pandemic, cardiac surgeries in patients with previous infection by COVID-19 were suspended or postponed, which led to surgeries performed in patients with an advanced stage of their disease and an increase in the waiting list. There is a heterogeneous attitude in Latin America on the optimal timing to cardiac surgery in patients with previous COVID-19 infection due to scarce data on its outcome. Two Latin American associations joined to establish common suggestions on the optimal timing of surgery in patients with previous COVID-19 infection.

**Methods:**

Data collection was performed using a pre-established form, which included year of publication, objective, type of study (prospective/retrospective, descriptive/analytical), number of patients, year of study, waiting time between infection and surgery, type of surgery, morbidity, mortality, and conclusions regarding the association between mortality and morbidity. Final recommendations were approved by the board of directors of Latin American Association of Cardiac and Endovascular Surgery (LACES) and Latin American Confederation of Anesthesia Societies (CLASA).

**Results:**

Of the initial 1,016 articles, 11 comprised the final selection. Only six of them included patients who underwent cardiac surgery. According to the analyzed literature, optimal timing for cardiac surgery needs to consider the following aspects: deferable surgery, symptomatic COVID-19 infection, completeness of COVID-19 vaccination.

**Conclusion:**

These recommendations derive from the analysis of the scarce literature published at present on outcomes after cardiac surgery in patients with previous COVID-19 infection. These are to be taken as a dynamic recommendation in which Latin American reality was taken into consideration.

**Table:** 

Abbreviations, Acronyms & Symbols	
**CLASA**	**= Latin American Confederation of Anesthesia Societies**
**COVID-19**	**= Coronavirus disease 2019**
**CT**	**= Computed tomography**
**EuroSCORE**	**= European System for Cardiac Operative Risk Evaluation**
**ICU**	**= Intensive care unit**
**LACES**	**= Latin American Association of Cardiac and Endovascular Surgery**
**PCR**	**= Polymerase chain reaction**
**SARS-CoV-2**	**= Severe acute respiratory syndrome coronavirus 2**

## INTRODUCTION

One of the central aspects of this coronavirus disease 2019 (COVID-19) pandemic period is the return of elective surgeries, especially those that had to be postponed because the patients sufered from the COVID-19 infection. In this situation, one of the main challenges is to assess the procedural time delay of these patients without compromising patient safety.

The time between the diagnosis of the infection and the proposed elective surgery seems crucial to assess the risk/beneft in making the necessary decisions to optimize the outcome of the surgery. A prospective, multicenter, international cohort study showed that mortality and respiratory complications were reduced in case of major surgeries when they were postponed at least seven weeks after infection^[1]^. Several societies^[2,3,4,5]^ have recommended to defer surgeries for four weeks but, in those considered major surgery cases, for more than seven weeks. In cases in which COVID-19 was severe, a thorough evaluation of the respiratory, cardiovascular, and immunological impacts is required.

Surgeries, in general, but specifically elective cardiac surgeries were especially affected during this period. High-volume services estimate a drop of procedures up to 50% in one year, in addition to a change in patient characteristics, that is, higher-risk patients who arrived to surgery later in the course of the disease, which was reflected by higher mortality^[6,7]^. The causes for the reduction in the number of surgeries are manifold: reduced number of beds in intensive care units (ICU), delay in consultations, strained healthcare budgets, and preoperative evaluation, among others.

Planning to reverse this situation and catch up with the surgical backlog requires a strategy that includes: 1) taking into account that the pathologies referred in cardiac surgery are time-sensitive — in other words, the natural evolution is the worsening of the patient’s clinical status with unfavorable outcome and reduced survival, which is why the previously proposed waiting times for major elective surgeries do not necessarily apply —; 2) taking into account that those patients with cardiovascular disease who sufered a COVID-19 infection were more likely to sufer more significant sequelae that conditioned the result of the surgery, therefore, it may require a re-evaluation to classify the risk better; and 3) the complexity of these procedures means that human and material resources are limited, so they must be used rationally.

To the difficulties inherent to this type of procedures, we must include the reality in Latin America, characterized by its heterogeneity and restricted healthcare budgets, which also limits decision-making. A study in seven Latin American countries whose objective was to analyze access to surgery in general shows important differences between these countries linked to socioeconomic differences and health models^[8]^.

Taking these considerations into account, the Latin American Association of Cardiac and Endovascular Surgery (LACES), in collaboration with the Cardiovascular Anesthesia Committee of the Latin American Confederation of Anesthesia Societies (CLASA), set themselves the objective of conducting a review of the available evidence regarding the period of delay necessary for the adequate recovery of COVID-19 patients before elective cardiac surgery. With the available information, we intend to make suggestions that consider the particular situation of Latin America, characterized by inequity in access to high complexity healthcare treatment, and the current epidemiological state of COVID-19, the incidence of Omicron variant coupled with elevated rates of vaccination.

## METHODS

The following databases were searched: PubMed®, Cochrane, Latin American and Caribbean Health Sciences Literature (or LILACS), and Scientific Electronic Library Online (or SciELO); no language restriction was applied.

Original articles corresponding to elective surgical procedures in adult patients who had previously sufered from COVID-19 infection were selected. The search was limited to articles published in 2021 and 2022 to include the most recent experience regarding the epidemiological situation of COVID-19.

Studies that evaluated only urgent and emergency surgeries, patients with infection at the time of surgery, and patients under 18 years of age were excluded.

The keywords used were based on the Medical Subject Headings (or MeSH) terms: surgical procedures; surgery; cardiac surgical procedures; COVID-19; COVID-19 vaccines, SARS-CoV-2; mortality; complications.

In the first stage, multiple terms combined by the OR operator were used.

The selection of the articles was carried out by two authors in the first instance independently. In case of discrepancies, a third reviewer was consulted, and their inclusion or not was decided by consensus. The procedure was carried out as follows: first, the articles’ titles and abstracts corresponding to the search were analyzed, selecting those that met the abovementioned criteria. The full-text of these articles was then obtained for analysis. And a manual search of the bibliographical references of the selected articles and related publications was carried out.

Data collection was performed using a pre-established form, which included: year of publication, objective, type of study (prospective/retrospective, descriptive/analytical), number of patients, year of study, waiting time between infection and surgery, type of surgery performed, morbidity, mortality, and conclusions regarding the association between mortality and morbidity.

The final recommendations were approved by the board of directors of LACES and CLASA.

## RESULTS

### Characteristics of the Studies

**Fig. 1:**
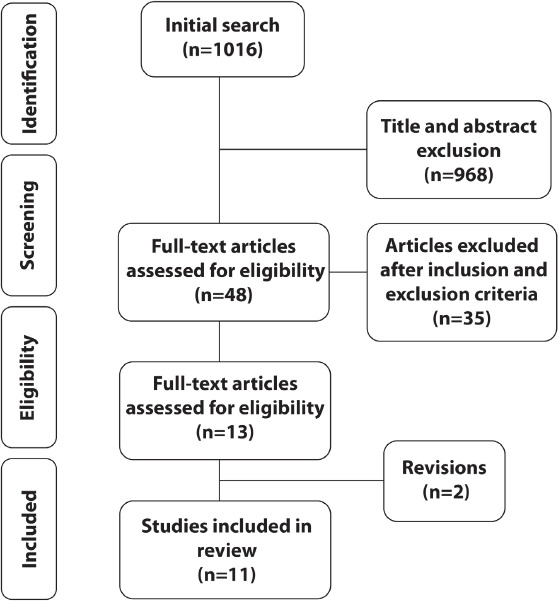
Preferred Reporting Items for Systematic Reviews and Meta-Analyses flowchart

Of the initially 1,016 articles whose title and abstracts were analyzed, 968 were excluded (Figure 1). The main reasons for rejection were: editorials or opinion articles, not including surgical patients, no information on mortality or morbidity, and data restricted to surgical outcomes of patients during active COVID-19 infection.

The remaining 48 articles were evaluated in their full-text. Of these, 35 were discarded because they corresponded to patients who underwent surgery in the context of the pandemic, but did not meet the criteria of having sufered from the infection *prior* to surgery, and two of them were revisions.

The 11 selected articles are shown in Table 1.

**Table 1:** Characteristics of included studies.

Author	Aim	Type of study	Number of patients	Study period	Type of surgery	Interval from COVID-19 and surgery	Morbidity related to COVID-19 infection	Mortality
COVID-19Surg^[1]^	To determine the optimal waiting time between COVID-19 infection and surgery	Prospective cohort, international, multicentric	3,127	October 2020	All types of surgery	0–2 weeks (1,138), 3–4 weeks (461), 5–6 weeks, and ≥ 7 weeks (1,202)	With > 7 weeks, similar morbidity to control group	With > 7 weeks, similar mortality to control group
Carrier^[7]^	Primary: characteristics and evolution of COVID-19-infected patients who required surgery Secondary: characteristics of patients who have recovered from infection and require surgery	Prospective cohort. Multicentric in Quebec, Canada	44	March-June 2020	All types of non-cardiac surgery	Does not discriminate. All patients were asymptomatic at the time of surgery or had more than 14 days of negative PCR	Complications were high in patients with COVID-19 at the time of surgery, especially in symptomatic patients. In recovered patients, morbidity was lower and similar between them. Events were similar with symptomatic COVID-19 and non-symptomatic COVID-19	It was higher in symptomatic COVID-19
Kho R^[9]^	Morbidity and mortality of a subgroup of surgeries postponed due to COVID-19	Prospective. Multicentric in the United States of America	114	July to December 2020	Gynecological surgery	Mean 98.3±64 days	The COVID-19-postponed subgroup showed no difference from the non-COVID-19 group	No data
Welk B^[10]^	Postoperative mortality in the early and late period after diagnosis of COVID-19-19	Retrospective. Database analysis in Ontario, Canada	146	February and May 2020	All types of surgery, including heart surgery	Within 14 days or later	Mortality was significantly lower in patients who underwent surgery 15-60 days after COVID-19 diagnosis	19.7% (£ 14 days) and 6.2% (after 15 days)
Nedelu M^[11]^	Postoperative complications	Retrospective. Multicentric. Europe.	35	June-October 2020	Bariatric surgery	Mean of 11.3 weeks (3-43 weeks)	No complications related to infection in the first 30 days	No mortality
VosburgW^[12]^	Morbidity and mortality in patients who had COVID-19 and recovered	Retrospective. Multicentric in the United States of America	53	No data	Bariatric surgery	54 days for asymptomatic and 102 days for mild symptoms. The average waiting time was 82 days.	No complications	No mortality
Gomez O^[13]^	Evolution of patients who presented COVID-19 in the perioperative period	Retrospective. Multicentric in Brazil	104	March 2020 – July 2021	Cardiac surgery	Mean 48 ±51 days before surgery in the group that had COVID-19 before surgery	Patients operated after 10 days of COVID-19 had lower morbidity	Surgery after 10 days of COVID-19 had lower mortality
Knisley^[14]^	To evaluate surgical results in patients with COVID-19	Retrospective in 2 centers	468	March – April 2020	Mainly gynecological and oncological	55.6% were diagnosed preoperatively	Higher incidence of serious complications and mortality with previous COVID-19	16.7% (with COVID-19) *vs.* 1.2% (not COVID-19). 23.5% (symptomatic) *vs.* 10.5% (asymptomatic)
Ismail^[15]^	Surgical results in patients with COVID-19	Single center. Retrospective	12	June 2020 – July 2021	Cardiac surgery	Average of 46 days	Higher incidence of non-invasive ventilation requirement	Non-major mortality
Baiocchi^[16]^	Surgical results in patients with a history of asymptomatic COVID-19	Retrospective	49	April 2020 – June 2020	Oncologic surgery	25-day average	No differences in postoperative complications	No operative mortality
Deng^[17]^	Surgical results in patients with COVID-19	Retrospective cohort	2858	March 2020 – May 2021	All types of surgery excluding emergencies. Vaccinated patients were not included	Peri-COVID-19 (0-4 weeks), early post-COVID-19 (5-8 weeks), and late post-COVID-19 (after 8 weeks) subgroups	Greater respiratory complications, pneumonia, thromboembolism, and sepsis in peri-COVID-19. Increased risk of pneumonia in early post-COVID-19	Does not report mortality

COVID-19=coronavirus disease 2019; PCR=polymerase chain reaction

Three of them corresponded to prospective articles^[1,7,9]^, and the rest were retrospective^[10,11,12,13,14,15,16,17]^). Among the prospective studies, the COVID-19Surg^[1]^ is the largest prospective registry and included all kinds of surgeries. The publication by Carrier et al.^[7]^ is a small prospective study of non-cardiac surgeries. From all the included studies, only six of them included patients who underwent cardiac surgery^[1,10,13,14,15,17]^. Among these, only Gomes et al.^[13]^ and Ismail et al.^[15]^ were specific for cardiac surgery.

### Analysis of the Studies

The COVID-19Surg Collaborative^[1]^ is one of the largest prospective registries of surgically treated patients with COVID-19. It consists of a multicenter prospective cohort with the participation of more than 100 countries between October and November 2020. Of a total of 140,231 patients, 2.2% had COVID-19. Most of these remained asymptomatic and were operated on within two weeks of diagnosis (36.4%) or after seven weeks (38.4%). When analyzing mortality and pulmonary complications compared to patients operated on without COVID-19, both were significantly higher in patients operated on before seven weeks of COVID-19 diagnosis. Restricting the analysis to only COVID-19 patients, mortality and respiratory complications were higher in patients with symptoms than in asymptomatic patients.

Carrier et al.^[7]^ evaluated the results of a population of patients operated on between March and June 2020, in Canada, through a prospective cohort study. Although they did not compare these patients with others without COVID-19, their results show higher mortality and complications in patients diagnosed with COVID-19, being worse in the symptomatic ones. When comparing with a group of symptomatic but non-COVID-19 patients (from other respiratory viral diseases), their data show similar results to symptomatic COVID-19 patients.

The SOCOVID-19 study^[9]^ is a multicenter prospective registry of patients undergoing gynecological surgery during the pandemic. Of 114 patients with a history of COVID-19, the incidence of serious complications and mortality was similar to those of patients without prior COVID-19. The average waiting time between COVID-19 diagnosis and surgery was 98 days.

Welk et al.^[10]^ is a retrospective study with data from the Ontario health system. Of the patients diagnosed with COVID-19 between February and May 2020, only 146 (0.6%) required surgical procedure. Most of these were trauma or plastic surgery. Operative mortality was the highest within 14 days of diagnosis. Two multicenter retrospective studies^[11,12]^ of patients with a history of COVID-19 undergoing bariatric surgery show similar results. In both studies, no cardiovascular or respiratory complications were reported in patients who sufered from COVID-19, with waiting times between infection and surgery that ranged between 21 and > 80 days.

Baiocchi et al.^[16]^ compared the postoperative evolution of 49 patients who underwent surgery. Of these, 49% were oncological surgeries. Only 22.9% of the patients had symptoms at the time of diagnosis of COVID-19, and in all of them, these were mild. There were no differences in postoperative complications, and operative mortality did not occur.

Deng et al.^[17]^ retrospectively evaluated multicenter data in the United States of America of patients with a previous diagnosis of COVID-19 operated from March 2020 to May 2021. They included all types of surgery and specifically excluded emergency surgeries and patients with prior severe acute respiratory syndrome coronavirus 2 (SARS-CoV-2) vaccination. Patients were grouped into “peri-COVID-19” (those operated on between 0-4 weeks after diagnosis), “early post-COVID-19” (5-8 weeks), and “late post-COVID-19” (after eight weeks). A total of 2,858 patients were included. Most of the included patients had a mild infection, and gynecological procedures were the most common procedure. The results showed that major elective surgery performed in the first four weeks was associated with a higher risk of complications, which persisted between four and eight weeks for pneumonia (two times higher risk). The risk was lower after eight weeks from the COVID-19 diagnosis.

### Studies Performed in Cardiac Surgery Patients

The study by Gomes et al.^[13]^ retrospectively evaluated the results of cardiac surgeries performed in patients with a previous diagnosis of COVID-19. Patients were classifed as having had COVID-19 > 10 days before surgery, within 10 days (before or after), or after surgery. Patients who underwent surgery > 10 days after COVID-19 diagnosis had a lower preoperative risk (lower European System for Cardiac Operative Risk Evaluation [EuroSCORE] II and lower percentage of emergency surgeries) and a higher rate of elective surgeries. The authors reported higher mortality and postoperative complications in patients with acquired COVID-19 within 10 days of surgery or after it. In the group of patients operated on for > 10 days from the diagnosis of COVID-19, the mean interval was 48 days, while for those operated on within 10 days, the mean was four days. It should be noted that the authors include a broad period of the pandemic, which extends from March 2020 to July 2021, composed of different epidemiological moments (different strains) as well as a different percentage of vaccination in the population.

Ismail et al.^[15]^ analyzed the results of a group of patients undergoing cardiac surgery with a history of COVID-19 before immunization. Although only 12 patients were included, their results show that after an average of 46 days between infection and surgery, operative mortality was similar to that of their patients without COVID-19, and an increased requirement for non-invasive ventilation was evidenced after extubation.

## DISCUSSION

The appearance of the SARS-CoV-2 pandemic has significantly impacted healthcare, thus delaying the resolution of surgical pathologies. Consequently, the list of patients awaiting surgery has increased, and, therefore, their stage in the natural history of the surgical disease is more advanced at the time of surgery. Two moments can be clearly seen in the evolution of the pandemic: one of high lethality, low incidence, and immunization of the population, and another of low lethality, high population incidence, and immunization. The second moment in Latin America seems to have been installed at the beginning of 2022. The prevalence of COVID-19 to date in some countries of Latin America is: 896,000 cases (25.8% of the population) in Uruguay, 9.06 million in Argentina (20% of the population), 3.54 million in Chile (18.5% of the population), and 30.3 million in Brazil (14.2%). A prevalence of up to 60% is estimated in communities with established transmission^[18,19]^. Such a prevalence means that surgery in patients with a history of COVID-19 infection will be an everyday situation.

In Table 1 we summarize the articles that analyze the surgical timing in patients who sufered from COVID-19. We need to highlight that the limitations to making recommendations on the cardiac surgery population based on current evidence are important due to several reasons: most of the data include a small percentage of cardiac surgical patients; most of the articles are retrospective and do not take into account the vaccination and/or epidemiological status of the population; evidence for Latin America is reported in only one article; in several reports, the waiting times are not clearly defined. Therefore, the recommendations in this document are mainly derived from low level of evidence and mostly from expert opinion. We acknowledge that new evidence will be released, and an update of the current document will be required.

From all the information collected, we can make the following generalizations:

- The postoperative evolution of patients with a history of asymptomatic COVID-19 is better than of those with symptomatic COVID-19.
- Patients operated on after seven weeks of COVID-19 diagnosis have a postoperative course similar to those who did not have COVID-19.
- Patients operated on before seven weeks were generally higher-risk patients, for which even after regression adjustments, selection bias still is the main limitation affecting the outcome data.
- Most of the information comes from the pre-Omicron era and with very low or no vaccination rate of the population.
- Most current recommendations focus on elective surgery, defined as surgery that can be deferred without significantly affecting patient survival.

However, we must consider that most patients who must undergo cardiac surgery correspond to time-sensitive surgery patients, that is, to an increased risk of morbidity and mortality if their surgery is postponed, so extrapolating the results of these studies to cardiac surgery requires careful evaluation.

Two studies report specific data from patients undergoing cardiac surgery. Gomes et al.^[13]^ provide data from a multicenter registry showing that patients operated on > 10 days of COVID-19 diagnosis (with a mean of 48 days) had a lower risk of complications and mortality. Operative mortality was 4.4%, which is similar to that reported by the same author in a national registry of coronary surgery (6.4%)^[20]^. It should be noted that, as expected, the patients operated on > 10 days after diagnosis were patients with lower risk (EuroSCORE II of 2.89) than those operated on within 10 days (EuroSCORE II of 4.93). Similarly, the percentage of emergency surgeries was significantly higher in the group of patients operated on within 10 days (45.9% *vs.* 13.3%) of diagnosis. Ismail et al.^[15]^ data, although coming from a smaller group of patients, point towards the same direction.

We believe the first step to consider due to its impact in the postoperative course is the degree of clinical impact during the COVID-19 infection^[1]^. In patients with indication for cardiac surgery, discrimination of symptoms due to COVID-19 or the natural progression of the disease is challenging and deserves careful evaluation with a multidisciplinary team to discriminate these aspects both from the clinical point of view and from complementary studies such as chest X-ray or computed tomography (CT) of the chest and/or spirometry.

Another issue that needs consideration is the state of complete vaccination. Clinical trials have shown that full vaccination (two doses of Pfzer or Moderna) effectively reduces the risk of complications associated with COVID-19 and mortality^[20,21]^. Prasad et al.^[22]^ retrospectively evaluated the postoperative outcomes of patients operated in the Veterans Hospitals in the United States of America from January 25 to March 25, 2022, and classifed them according to immunization status. Complete vaccination was defined as those who received at least two doses of Pfzer or Moderna at least 14 days before surgery. The authors demonstrated that fully vaccinated patients were associated with fewer postoperative respiratory and thromboembolic complications, shorter hospital stay, and a lower risk of acquiring postoperative COVID-19. These results were seen in the subgroup of patients without previous COVID-19 infection. However, the beneft could not be assessed in the subset of patients with previous COVID-19 due to the small number of patients.

Due to the scant evidence regarding the ideal timing for cardiac surgery, most recommendations will be based on expert opinion. This takes on even greater relevance in the current context of the pandemic characterized by the circulation of a viral subtype with lower lethality, greater contagiousness, and a very high vaccination rate in a group of patients who should be considered high risk since they have time-sensitive pathologies. Taking a restrictive stance regarding the surgical coordination of patients with an indication for cardiac surgery has the risk of significantly increasing waiting times with the consequent increase in mortality. This must be balanced against the fact that taking a lax and early posture could increase postoperative complications and, eventually, operative mortality. Faced with this situation, it is imperative to adapt the existing evidence to issue an expert opinion and assist Latin American colleagues in decision-making. To make recommendations, our group considered especially issues related to health equity. As we have mentioned, healthcare delivery in Latin America is highly heterogeneous. Long waiting times and restrictive recommendations will mainly affect countries with accessibility strain, deepening, and, therefore, inequity in healthcare delivery in Latin America. Balancing equity without increasing the risk for postoperative complications was the challenge we faced while writing the current statement. The comparison between LACES/CLASA recommendations and other societies is shown in Table 2.

**Table 2:** Comparison between LACES/CLASA recommendations and other societies.

	American Society of Anesthesiology	Association of Anesthesiologists	LACES/CLASA
Asymptomatic or mild symptoms	4 weeks	According to the risk of the surgery and the patient	2 weeks or 4 weeks depending on vaccination
Symptomatic	6 weeks (10 weeks in diabetic or immunocompromised patients)	Minimum 7 weeks	4 or 7 weeks depending on vaccination
Admission to ICU	12 weeks	Does not distinguish	7 weeks
Vaccination	Does not distinguish	Does not distinguish	Distinguish
Publish date	December 8, 2020	22 February 2022	
Reference	4	3	

ICU=intensive care unit; LACES/CLASA=Latin American Association of Cardiac and Endovascular Surgery/Latin American Confederation of Anesthesia Societies

### Algorithm Proposal for Cardiac Surgery

**Fig. 2:**
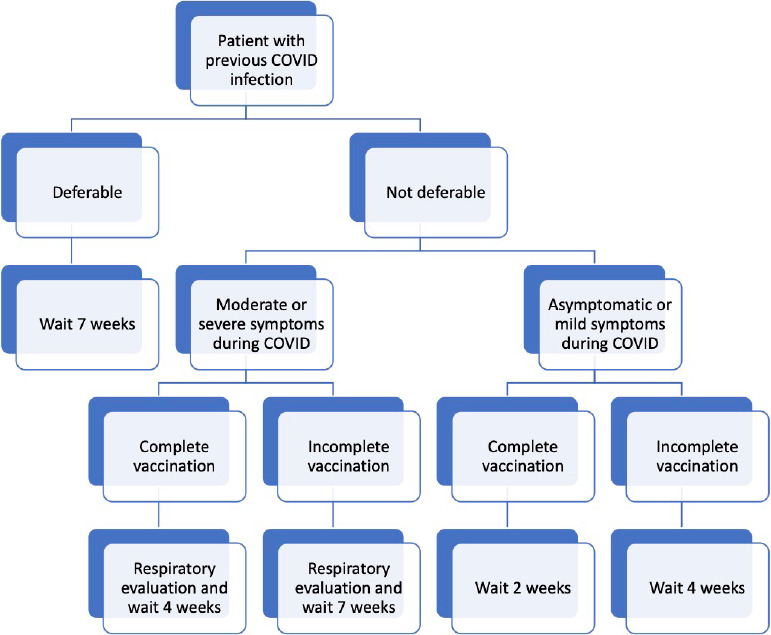
Recommendation algorithm for timing of cardiac surgery in patients with previous coronavirus disease 2019 (COVID-19) infection.

The algorithm proposed by LACES and CLASA for managing patients with an indication for cardiac surgery with a *previous* infection of COVID-19 is shown in Figure 2.

All patients, before cardiac surgery, must have a polymerase chain reaction test for SARS-CoV-2 and be considered cured of their COVID-19 infection. The latest positive test for SARS-CoV-2 should be considered as time 0.

1. The first decision should be whether the patient’s condition represents a deferrable surgery or not.
  We define as deferrable surgery a condition in which the delay of > 7 weeks in its resolution does not increase the risk of death or postoperative complications. We consider deferrable surgery the following conditions: interatrial septal defect, stable coronary artery disease, stable aortic aneurysm, and stable valve disease (severe asymptomatic disease with preserved ventricular function)^[19]^.
  If the patient is deferrable, our group recommends waiting seven weeks for surgery.
2. **Not deferrable**
  Two situations should be considered:
**a) Asymptomatic or Mild Symptoms**
    We consider asymptomatic patients and those with mild symptoms to belong to the same category. Mild symptoms refer to: cough, odynophagia, otalgia, and clinical elements that suggest involvement mainly of the upper respiratory system. Any other symptom that suggests systemic involvement will be considered moderate or severe (headache, fever, dyspnea, hospitalization, oxygen desaturation).
    The next step is vaccination status.
    Complete vaccination is defined as those who received at least two doses of Pfzer or Moderna at least 14 days before surgery. We are aware that several Latin American countries have based their vaccination on other vaccines. Unfortunately, there is no evidence on their effectiveness on surgical patients and, therefore, its adequacy should be evaluated at an individual basis by a multidisciplinary team.
**- Complete Vaccination**
    Proceed to surgery 14 days after diagnosis. These patients should be managed as we currently work-out patients with any viral upper respiratory infection before surgery.
**- Incomplete Vaccination (or No Vaccination)**
    Wait four weeks for surgery. In these cases, evidence has shown that complete vaccination is associated with better outcomes. Therefore, completing the vaccination schedules should be considered before surgery.
**b) Moderate to Severe Symptoms**
**- Complete Vaccination**
    A respiratory evaluation should be performed. Although a chest CT scan is the ideal evaluation, access may be difficult in different countries. Therefore, our group recommends a chest X-ray and spirometry before surgery as a minimum requirement. If no alterations are noted after multidisciplinary evaluation, surgery is recommended after four weeks of COVID-19.
**- Incomplete Vaccination (or No Vaccination)**
    Evaluation should proceed as in the case of complete vaccination. If no alterations are noted after multidisciplinary evaluation, surgery is recommended after seven weeks of COVID-19. As mentioned before, completing the vaccination schedules should be considered before surgery.
**c) Patients who Required ICU**
    A multidisciplinary team should evaluate this group of patients to decide whether their cardiac clinical status permits proceeding to surgery after seven weeks of being discharged from ICU, irrespective of the vaccination status. During this time, completing vaccination at least 14 days before surgery should be recommended.

## CONCLUSION

We are faced with a unique historical situation. Massive deferrals of surgical patients due to COVID-19 pandemic. Much is unknown regarding the best way to manage patients with previous COVID-19 infection. This is especially true for cardiac surgical patients in whom the progression of their disease intricates with COVID-19 symptoms and may even accelerate the natural history of their disease. The reality of Latin America is much different from the ones of the United Kingdom, Europe, or the United States of America. Therefore, LACES and CLASA believe there is an urgent need to guide our physicians on a common road of action, striving to decrease the risk of postoperative complications and progression of the cardiac disease and avoid deepening health inequity. Considering the scarce evidence, these recommendations are mainly expert opinions and will be under revision as new evidence emerges.
